# Interactions of SARS Coronavirus Nucleocapsid Protein with the host cell proteasome subunit p42

**DOI:** 10.1186/1743-422X-7-99

**Published:** 2010-05-17

**Authors:** Qin Wang, Chuan Li, Quanfu Zhang, Tao Wang, Jiandong Li, Wuxiang Guan, Jianshi Yu, Mifang Liang, Dexin Li

**Affiliations:** 1 State Key Laboratory for Molecular Virology and Genetic Engineering, National Institute for Viral Disease Control and Prevention, China CDC 100 Ying Xin Jie, Xuan Wu Qu, Beijing 100052, China; 2 State Key Laboratory for Infectious Disease Control and Prevention, National Institute for Viral Disease Control and Prevention, China CDC 100 Ying Xin Jie, Xuan Wu Qu, Beijing 100052, China

## Abstract

**Background:**

Severe acute respiratory syndrome-associated coronavirus (SARS-CoV) spreads rapidly and has a high case-mortality rate. The nucleocapsid protein (NP) of SARS-CoV may be critical for pathogenicity. This study sought to discover the host proteins that interact with SARS-CoV NP.

**Results:**

Using surface plasmon resonance biomolecular interaction analysis (SPR/BIA) and matrix-assisted laser desorption/ionization time of flight (MALDI-TOF) mass spectrometry, we found that only the proteasome subunit p42 from human fetal lung diploid fibroblast (2BS) cells bound to SARS-CoV NP. This interaction was confirmed by the glutathione S-transferase (GST) fusion protein pulldown technique. The co-localization signal of SARS-CoV NP and proteasome subunit p42 in 2BS cells was detected using indirect immunofluorescence and confocal microscopy. p42 is a subunit of the 26S proteasome; this large, multi-protein complex is a component of the ubiquitin-proteasome pathway, which is involved in a variety of basic cellular processes and inflammatory responses.

**Conclusion:**

To our knowledge, this is the first report that SARS-CoV NP interacts with the proteasome subunit p42 within host cells. These data enhance our understanding of the molecular mechanisms of SARS-CoV pathogenicity and the means by which SARS-CoV interacts with host cells.

## Background

The outbreak of severe acute respiratory syndrome (SARS), which began in the Guangdong Province of China, spread rapidly to more than 30 countries during 2003. SARS has an acute onset, is highly transmissible and has a high case-mortality rate (approximately 10%) [1,2]. During SARS infection, three phases of viral replication result in respiratory tract pathological changes and an over-exuberant host immune response. This mediates immunopathological damage of the lungs and other organs, and pulmonary fibrosis. SARS mortality is caused primarily by extensive lung damage and severe lymphopenia [3]. Approximately 10% of individuals (6.8% of patients younger and 55% of patients older than 60 years of age) with clinical symptoms died as a consequence of immunopathological lung damage, caused by a hyperactive antiviral immune response [4].

The mechanism of the serious damage to the respiratory system caused by SARS-CoV remains unclear. At least two possibilities exist: (i) direct damage to cells and tissues by the SARS-CoV and (ii) indirect damage, mediated primarily by the cellular immune response and cytokines.

SARS-CoV nucleocapsid protein (SARS-CoV NP) is an extensively phosphorylated, highly basic, vital structural protein the primary function of which is to form a helical ribonucleoprotein complex with viral RNA (vRNA). This complex comprises the core structure of the SARS-CoV virion. A variety of functions have been ascribed to SARS-CoV NP, including packaging, transcription, and replication. However, these are based solely on known functions of the NP of other coronaviruses [5]. SARS-CoV NP shows intrinsic multimerization and interacts with M protein, suggesting that NP is both critical to formation of the viral nucleocapsid core and is involved in virion assembly [6,7].

Sequence analysis indicates that the RNA-binding domain of SARS-CoV NP may be located at residues 178-205 [8]. Motif scanning predicted a bipartite nuclear localization signal, located at residues 373-390, suggesting that this protein may play a role in the pathogenicity of SARS-CoV [9].

SARS-CoV NP is highly immunogenic. Antibodies against the nucleocapsid protein are longer lived and occur in greater abundance in SARS patients than antibodies against other viral components such as the spike, membrane and envelope proteins [10]. This may be due to the presence of higher levels of nucleocapsid protein, compared with other viral proteins, after SARS-CoV infection [11]. These data suggest that the SARS-CoV NP is strongly antigenic and so may play an important role in generation of the host immune response and immunopathological damage.

In this study, SPR/BIACORE, MALID-TOF MS, the GST-fusion expression pulldown technique, and cell co-localization were used to investigate the interactions of SARS-CoV NP with host cell proteins. In this way, we sought to further elucidate the molecular pathogenic mechanisms of SARS-CoV. This, in turn, will allow development of novel therapeutics effective against this debilitating infection.

## Materials and methods

### Plasmids and bacterial strains

Plasmid pET22b-SNP22b was constructed by cloning the SARS-CoV NP (SNP22b) gene by reverse transcriptase PCR (RT-PCR) using vRNA from SARS-CoV SCV-8 (isolated from a SARS patient in Beijing, China) with the following primers: forward: 5'-GAAGGATCCGATGTCTGATAATGGACCCCAATCAA-3', reverse: 5'-GCTGAATTCTTAATGGTGATGGTGATGGTGTGCCTGAGTTGAATCAGCAGAAGC-3'. PCR products were purified and inserted into the pET22b plasmid using BamHI/EcoRI. The p42 gene was amplified by RT-PCR using mRNA from 2BS cells with the following primers: p42 forward: 5'-GATGAATTCATGGCGGACCCTAGAGATAAGG-3', reverse: 5'-GATCTCGAGTTACACAGGTTTGTAGTCCAATTTAG-3'. PCR products were purified and cloned into the pGEX-5X-1 plasmid (Pharmacia, GE) using XhoI/EcoRI, to generate pGEX-5X-1-p42. The plasmid pcDNA3.0-SNP22b was constructed by subcloning the SNP gene, released from pET22b-SNP22b by BamHI/EcoRI, into pcDNA3.0 (Invitrogen). All of the recombinant clones were confirmed by sequencing. *Escherichia coli *DH5α and BL21 (DE3) were obtained from Invitrogen (USA).

### Cell culture, transfection, and reagents

Human fetal lung diploid fibroblast (2BS) cells (SARS-CoV susceptible), were obtained from ATCC (USA) and maintained in DMEM (Invitrogen), containing 10% fetal bovine serum (FBS) and gentamicin (5 μg/mL). Transfection of 2BS cells was carried out using Lipofectamine 2000 according to the manufacturer's protocol. All restriction endonucleases were purchased from New England Biolabs (UK). Horse anti-SARS-CoV NP polyclonal antibody was a gift from the Academy of Military Medical Sciences. The anti-SARS-CoV NP mAb was prepared in our laboratory, while goat anti-mouse HRP-IgG and goat anti-rabbit IgG-FITC conjugates were purchased from Sigma (USA).

### ELISA assay

Microtiter plates were coated with anti-SARS-CoV NP mAb (5 μg/mL) in bicarbonate buffer (15 mM Na_2_CO_3_, 35 mM NaHCO_3_, pH 9.6) overnight at 4°C. Plates were washed with PBST (100 mM NaCl, 10 mM Na_2_HPO_4_, 3 mM KH_2_PO_4_, 0.05% (v/v) Tween 20, pH 7.2) and blocked using 5% (w/v) fat-free milk in PBST for 1 h at 37°C. Plates were then washed with PBST and increasing concentrations of SARS-CoV NP added. SARS-CoV NP, bovine serum albumin (BSA), and PBS were the positive, negative, and blank controls, respectively. All plates were then incubated at 37°C for 1 h, followed by washing six times with PBST. All wells were incubated with anti-SARS-CoV NP horse polyclonal antibody-HRP conjugate for 1 h at 37°C. Plates were subsequently washed six times with PBST; TMB chromogenic substrate solution and stop solution were then added and the A_450 _was determined.

### Immunoblot analysis

Samples were lysed in 1× loading buffer (0.08 M Tris, 2.0% (w/v) SDS, 10% (v/v) glycerol, 0.1 M dithiothreitol, 0.2% (w/v) bromophenol blue, pH 6.8). Samples were boiled for 10 min and resolved by one-dimensional SDS-PAGE. Proteins were transferred onto nitrocellulose membranes and the membranes were probed with the appropriate primary antibody. Secondary antibodies were alkaline phosphatase-conjugated anti-human, anti-rabbit, anti-mouse, or anti-goat IgG (Jackson Immunoresearch, Inc.). Gels were stained using 5-bromo-4-chloro-3-indolyl phosphate (BCIP) and nitro blue tetrazolium (NBT) solutions (Sigma).

### Expression, identification, and purification of recombinant SARS-CoV NP

Plasmid pET22b-SNP22b was transformed into *E*. *coli *BL21. The fusion protein SNP-His was expressed under 1 mM IPTG at 22°C for 12 h. Bacteria were harvested and lyzed in lysis buffer (1 mg/mL lysozyme (Sigma), 1% (v/v) Triton X-100, 5 μg/mL DNAse, 5 μg/mL RNAse) at 4°C for 30 min. Lysates were harvested by centrifugation (3,000 × *g*, 30 min, 4°C). SARS-CoV NP levels in supernatants were determined by Western blotting using anti-SARS-CoV NP horse polyclonal IgG as the primary antibody.

SARS-CoV NP was purified by affinity chromatography using His. Bind^® ^Resin (Novagen) and ion exchange chromatography, using the Econo pac High CM cartridge (Bio-Rad). Purified NP was desalted using a PD-10 desalting column (Amersham) and concentrated by ultrafiltration using Centricon™ centrifugal filters (10 kDa MWCO, Millipore). Purified NP was suspended in HBS-EP buffer (pH 7.4, appropriate for BIAcore). SARS-CoV NP concentration was determined using a BCA protein assay reagent kit (Pierce). SARS-CoV NP activity was detected by indirect ELISA with anti-SARS-CoV NP horse polyclonal antibody.

### Identification of SARS-CoV NP-binding host cellular proteins

2BS cells were washed twice in cold 1× PBS and harvested. Pellets were lysed in 700 μL lysis buffer (1% Nonidet P40 and 20 μL protease inhibitor cocktail set III (Calbiochem) in 1 mL HBS-EP Buffer (pH 7.4, BIAcore), followed by freezing at -70°C and thawing at room temperature three times. Cells were harvested by centrifugation (12,000 × *g*, 20 min, 4°C). Supernatant was collected, aliquoted, and stored at -70°C.

Host cell protein capturing was performed on a Sensor Chip CM5 (Amersham Bioscience) using BIAcore3000 and the amine coupling direct capture method, as described by the manufacturer. Briefly, SARS-CoV NP was diluted in 10 mM NaAc (pH 5.5). A Sensor Chip CM5 flow cell was activated with 70 μL of a mixture of EDC/NHS (Amine Coupling Kit, Amersham Bioscience), SARS-CoV NP (70 μL, 80 μg/mL) injected, and the flow cell was blocked using ethanolamine (70 μL, 1 M). Host cell lysate was then injected and allowed to flow through the cell containing immobilized SARS-CoV NP. A flow cell immobilized with HBS-EP buffer through which an identical volume of host cell lysate flowed represented the negative control. Lysis buffer, injected into a flow cell immobilized with SARS-CoV NP, functioned as the blank control. The reaction unit (RU) of SARS-CoV NP is obtained thus: RU_reaction _- RU_blank control _- RU_negative control _= RU_SARS-CoV NP_

Captured proteins were precipitated by addition of a three-fold volume of cold acetone at -20°C overnight. Pellets were dissolved in HBS-EP buffer (10 μL) and 2× loading buffer (10 μL) and then resolved by 1-D SDS-PAGE on a 12% gel. The gel was stained using modified Neuhoff's colloidal Coomassie blue G-250 stain solution (0.12% (w/v) Coomassie Blue G-250 dye, 10% (w/v) ammonium sulfate, 10% (v/v) phosphoric acid, 20% (v/v) methanol) [12].

Protein bands of interest were cut out of the gel and rinsed twice in 50% (v/v) methanol (HPLC grade). In-gel digestion was performed using sequencing-grade modified trypsin (Promega). Extracted peptides were analyzed by matrix-assisted laser desorption ionization time-of-flight (MALDI-TOF) mass spectrometry by the Life Science Academy of Beijing University, China using an Ultraflex™ MALDI-TOF/TOF (Bruker) and Data Explorer (v. 4.0). Proteins were identified by comparison of their monoisotopic masses with those in the NCBI nonredundant or SwissProt databases using the MS-Fit search engine of ProteinProspector.

### Pulldown assay

*E*. *coli *BL21 were transformed with the pGEX-5X-1-p42 plasmid. The fusion protein p42-GST was expressed by addition of IPTG (1 mM) at 22°C for 12 h. *E. coli *were lysed in lysis buffer (20 mM Tris-HCl (pH 8.0), 200 mM NaCl, 1 mM EDTA (pH 8.0), 0.5% NP40). SDS-PAGE and immunoblotting (anti-GST mAb, Pharmacia) were used to identify soluble expression of the p42-GST fusion protein.

Glutathione Sepharose 4B beads (Pharmacia, GE) were suspended in lysis buffer to a concentration of 50% (w/v). Lysates of *E*. *coli *expressing the p42-GST fusion protein (1 mL), lysates of *E*. *coli *expressing GST alone, and lysis buffer represented the test sample, negative control, and blank control, respectively. Glutathione Sepharose 4B beads and bacterial lysates were added into three tubes, mixed for 60 min at 4°C, and 40 μL of this mixture was saved for SDS-PAGE analysis. The remaining mixture was washed with lysis buffer three times. Pellets were suspended in lysis buffer (200 μL), to which was added purified SARS-CoV NP (100 μL) and the mixture was further incubated at 4°C. After 3 h, the reaction mixture was washed three times in cold lysis buffer and eluted by boiling in 2× loading buffer. Eluted materials were subsequently analyzed by immunoblotting.

### Immunofluorescent (IF) assay

2BS cells were cultured on coverslips in 6-well plates to 70-80% confluence and transfected with plasmid pcDNA3.0-SNP22b using the lipofectAMINE™ 2000 transfection kit (Invitrogen). At 48 h after transfection, coverslips were washed in 1× PBS and fixed in 1% paraformaldehyde. Coverslips were then blocked with 5% (w/v) skimmed milk at 37°C for 60 min and washed six times in 1× PBS. Following incubation with a 1:100 dilution of mouse anti-SARS-CoV NP mAb and a 1:100 dilution of anti-p42 polyclonal antibody (Biomol, USA) at 37°C for 60 min, coverslips were washed six times with 1× PBS. After incubation with a 1:80 dilution of goat anti-rabbit IgG-FITC (Sigma) and a 1:200 dilution of goat anti-mouse IgG-TRITC (Sigma) conjugates at 37°C for 45 min, coverslips were again washed six times in distilled water and air-dried before being mounted on a slide with interspaces containing 50% (v/v) glycerol. Photomicrographs were taken using a confocal microscope (Confocal Microscope FluoView™ FV1000).

## Results

### Expression, identification and purification of SARS-CoV NP

**Figure 1:**
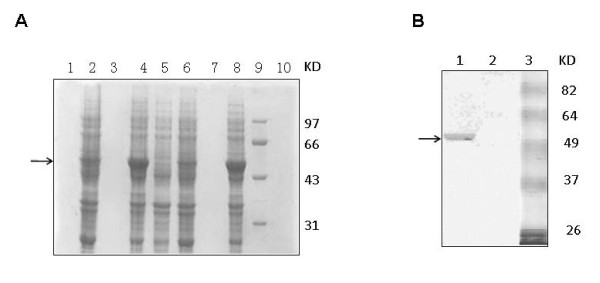
**Expression and Identification of SNP22b (SARS-CoV NP)**. (**A**) Supernatants (lanes 1 and 10) and pellets (lanes 2 and 6) of *E. coli *BL21 containing the pET22b-SNP22b vector expressing SARS-CoV NP, without IPTG induction. Supernatants (lanes 3 and 7) and pellets (lanes 4 and 8) of *E. coli *BL21 containing the pET22b-SNP22b vector and expressing SARS-CoV NP, induced with IPTG. Pellets of *E*. *coli *BL21 containing pET22b induced with IPTG (lane 5). Molecular weight markers (lane 9). The position of SARS-CoV NP (~47 kDa) is indicated by an arrow. (**B**) Western blot analysis of SARS-CoV NP using an anti-SARS-CoV NP horse polyclonal antibody. Lysate of *E*. *coli *BL21 expressing SARS-CoV NP, induced by IPTG (lane 1). Lysate of *E*. *coli *BL21 containing pET22b induced by IPTG (lane 2). Molecular weight markers (lane 3). The position of SARS-CoV NP (~47 kDa) is indicated by an arrow.

A plasmid expression vector, pET22b-SNP22b, was constructed to express SARS-CoV NP in *E*. *coli *BL21. Expression of SARS-CoV NP was confirmed by SDS-PAGE (Fig. 1A) and Western blotting using a horse polyclonal antibody against SARS-CoV NP (Fig. 1B). When expression was induced using isopropyl β-D-thiogalactopyranoside (IPTG), cells transfected with pET22b-SNP22b produced large amounts of SARS NP, amounting to up to 2% of total soluble protein (Fig. 1A; lanes 4 and 8).

Purification of SARS-CoV NP was achieved first by Ni^2+ ^chelate affinity chromatography, where SARS-CoV NP was eluted using wash buffer containing imidazole (250 mM), and then further purified by cation exchange chromatography. The purity of SARS-CoV NP achieved by means of this procedure was greater than 90%. SARS-CoV NP was desalted, dissolved in HBS-EP buffer, concentrated, and quantified. Indirect ELISA data suggested that purified SARS-CoV NP had a specific antibody binding activity similar to the native form (data not shown).

### Capture of proteasome subunit p42 by SARS-CoV NP

**Figure 2:**
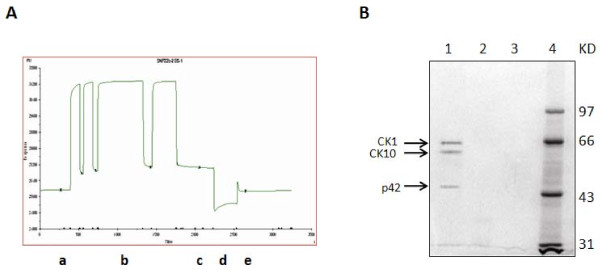
**Identification of SARS-CoV NP-associated cellular protein(s)**. (**A**) Interaction of SARS-CoV NP with 2BS cell lysate proteins. The maximum reaction intensity was 1120 RU (manual injection). Baseline (a), injection of host cell lysate (b), RU value of flow cell after injection of host cell lysate (reactive amount = RU_c_-RU_a_) (c), eluted and recovered captured proteins (d), regeneration of flow cell (e). (**B**) Proteins captured from 2BS cell lysate (lane 1), blank controls (lane 2), and negative controls (lane 3). Molecular weight markers (lane 4). Arrows indicate the position of captured proteins.

SARS-CoV NP was diluted to 140 μg/mL in 10 mM NaAc (pH 5.5) for immobilization. Regeneration was achieved using NaOH (50 mM). Immobilization of SARS-CoV NP in a sensor chip CM5 flow cell resulted in an RU value of 12355.1. This increased to 31 after HBS-EP buffer was immobilized in an adjacent flow cell of the same chip and increased further upon addition of 2BS cell lysates to the flow cell containing SARS-CoV NP. A stable level (1120 RU) was achieved after a total of 70 μL 2BS cell lysate was injected. The flow cell was then washed six times and NaOH (5 μL, 50 mM) was injected to elute proteins bound to SARS-CoV NP. The RU values of the blank and negative controls were 23 RU and 41 RU, respectively. Thus, the specific reaction of SARS-CoV NP and 2BS cell lysate was quantified as 1056 RU (calculated by 1120RU-23RU-41RU), suggesting that SARS-CoV NP had bound to at least one protein present in 2BS cell lysate (Fig. 2A). Captured proteins were recovered and collected by repeating the direct capture and blank control procedures.

The protein(s) captured by SARS-CoV NP were precipitated, resolved by 1D SDS-PAGE and visualized using modified Neuhoff's colloidal Coomassie blue G-250 staining. SARS-CoV NP was found to have bound three proteins from 2BS cell lysate, the molecular weights of which ranged from 43 to 66 kDa (Fig. 2B, lane 3). No protein was captured from the blank or negative controls (Fig. 2B, lanes 1, 2). Analysis by MALID-TOF mass spectrometry identified the proteins as: 26S proteasome regulatory subunit S10B (proteasome subunit p42 or proteasome 26S subunit ATPase 6, P62333; Fig. 2B), cytokeratin 1 (CK1, P04264), and cytoskeletal protein 10 (CK10, P13645). Cytokeratins are a subfamily of intermediate filament proteins. Cytokertin 1 (CK1) has the highest molecular weight and the highest isoelectric point of the family, while cytokeratin 10 has the lowest molecular weight and a low isoelectric point. These proteins are expressed in combinations that vary according to the type of epithelium within which they are found. Proteasome subunit p42 was selected for further assessment; the roles of CK1 and CK10 will be considered in a subsequent study.

### Interaction of proteasome subunit p42 with SARS-CoV NP *in vitro*

**Figure 3:**
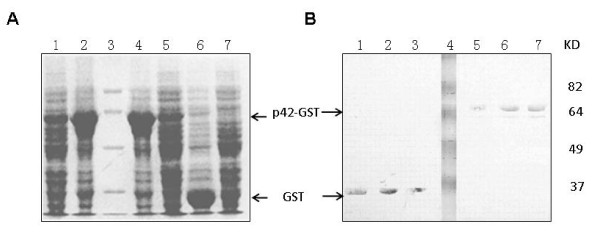
**Expression and identification of GST and the p42-GST fusion protein**. (**A**) Lysate of *E*. *coli *BL21 containing the pGEX-5X-1-p42 vector, expressing p42-GST fusion protein without IPTG induction (lanes 1, 5) or induced by IPTG (lanes 2, 4). Lysate of *E*. *coli *BL21 containing the pGEX-5X-1 vector expressing GST with IPTG induction (lane 6) or without IPTG (lane 7). Molecular weight markers (lane 3). (**B**) Western blot analysis of p42-GST fusion protein and GST expression using an anti-GST mAb. Lysates of GST-expressing *E*. *coli *BL21 with IPTG induction (lanes 1, 2). Lysates of p42-GST fusion protein-expressing *E*. *coli *BL21 with IPTG induction (lanes 6, 7). Supernatants of *E*. *coli *BL21 expressing GST lysed using lysozyme (lane 3). Supernatants of *E*. *coli *BL21 expressing p42-GST fusion protein lysed by lysozyme (lane 5). Molecular weight markers (lane 4).

**Figure 4:**
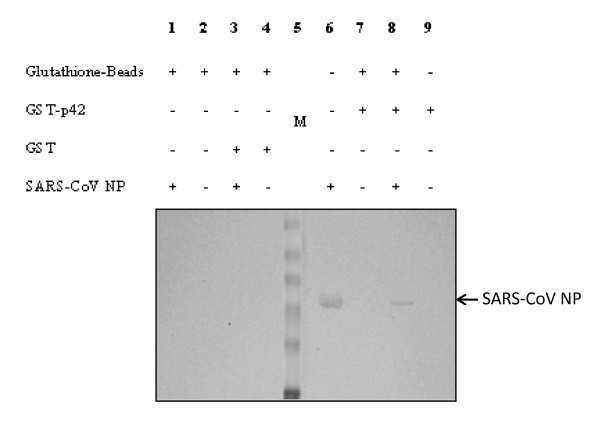
**Western blot analysis of the interaction between p42-GST fusion protein and SARS-CoV NP *in vitro***. Bead + lysis buffer + NP (lane 1), bead + lysis buffer (lane 2), bead + GST lysate + NP (lane 3), bead + GST lysate (lane 4). Molecular weight markers (lane 5). SARS-CoV NP-SNP22b (positive control; lane 6), bead + p42-GST lysate (lane 7), bead + p42-GST lysate + NP (lane 8), p42-GST fusion protein bacterial lysate (lane 9). The position of SARS-CoV NP is indicated by an arrow.

To examine further the interaction between SARS-CoV NP and proteasome subunit p42 *in vitro*, GST and the p42-GST fusion protein were produced, purified, and identified by 1D SDS-PAGE (Fig. 3A) and Western blotting, using an anti-GST mAb (Fig. 3B). Interaction of SARS-CoV NP with the proteasome subunit p42 *in vitro *was identified using the p42-GST fusion protein pulldown technique. Data suggested that the SARS-CoV NP was pulled down by p42-GST fusion protein (Fig. 4, lanes 6, 8), but not by either of the negative controls: GST alone (Fig. 4, lanes 1, 3, 5, 7) or glutathione Sepharose 4B beads (Fig. 4, lanes 2, 9). Thus, these data suggest that SARS-CoV NP exhibits a specific interaction with the proteasome subunit p42 *in vitro*.

### Co-localization of SARS-CoV NP and proteasome subunit p42 in 2BS cells

**Figure 5:**
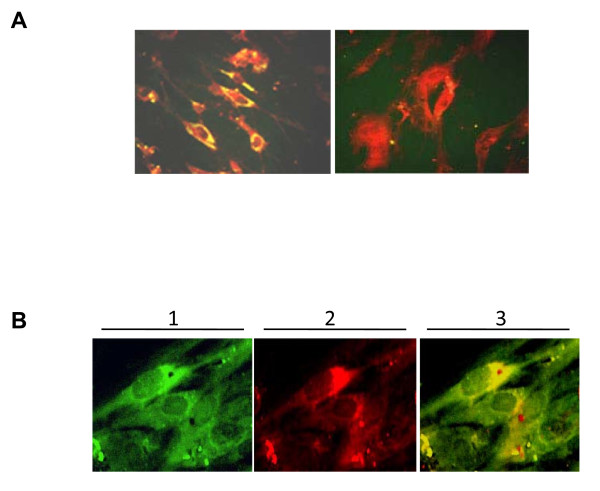
**Co-localization of SARS-CoV NP and p42 in 2BS cells**. (**A**) SARS-CoV NP was expressed in 2BS cells transfected with recombinant -pcDNA3.0+SNP22b (left panel), 2BS cells transfected with pcDNA3.0 (right panel; negative control). (**B**) Proteasome subunit p42 in 2BS cells localized with anti-p42 antibody and anti-rabbit IgG-FITC conjugate, excitation at 488 nm (lane 1). SARS-CoV NP expressed in 2BS cells, localized using an anti-SARS-CoV NP mAb and anti-mouse IgG-TRITC conjugate, excitation at 568 nm (lane 2). Co-localization was detected by merging lanes 1 and 2 (lane 3).

To analyze the interaction of SARS-CoV NP and proteasome subunit p42 within host cells, SARS-CoV NP was expressed in 2BS cells and identified by indirect immunofluorescence using an anti-SARS-CoV NP mAb (Fig. 5A). After transfection of 2BS cells with SNP22b-pcDNA3.0, the proteasome subunit p42 was detected by means of an anti-p42 antibody and anti-rabbit IgG-FITC conjugate (Fig. 5B, lane 1; excitation wavelength 488 nm). Expression of SARS-CoV NP in 2BS cells was confirmed using an anti-SARS-CoV NP mAb and anti-mouse IgG-TRITC conjugate (Fig. 5, lane 2; excitation wavelength 568 nm). Thus, the co-localization signals (Fig. 5, lane 3; excitation by both 488 nm and 568 nm) of p42 and SARS-CoV NP in 2BS cells were elucidated by merging lane 1 and lane 2 of Fig 4. This usually occurs when fluorescently labeled molecules bind to targets in close proximity.

## Discussion

SARS is an emerging infectious disease that has become a global public health concern in the 21^st ^century. However, the pathogenesis of SARS remains unclear. It has been suggested that the cytokine storm observed during the early stages of SARS in most patients contributes to the progression of systemic inflammatory response syndrome [13]. The SARS-CoV NP has been shown to be highly immunogenic and the predominant target for the humoral immune response during infection with SARS-CoV [14]. This process may trigger cytokine production and so induce apoptosis in host cells [13].

Law suggested that SARS-CoV, during infection of dendritic cells, evades the immune response by down-regulating expression of the anti-viral chemokines IFN-α, β and γ and IL-12p40, while simultaneously up-regulating that of others: for example, TNF-α, IL-6, MIP-1α, and IP-10 [15]. Additionally, it seems likely that proinflammatory cytokines released by macrophages in pulmonary alveoli play an important role in the pathogenesis of SARS.

SARS-CoV is capable of inducing apoptosis of Vero E6 cells *in vitro *[16]. Apoptosis of T- and B-lymphocytes in the immune organs and pulmonary alveoli and mononuclear inflammatory infiltration in the lungs, lymph nodes, and spleens of SARS-CoV infected individuals has been observed. Some SARS-CoV proteins, for example, E protein, NP, ORF7a, ORF3a, and ORF3b, have been shown to induce apoptosis *in vitro *[13,17-19]. Thus, the rapid apoptosis induced by SARS-CoV may be one of the mechanisms responsible for the damage to the lungs and immune organs observed in SARS patients.

Interaction of viral and host proteins may contribute to the processes involved in viral infection, replication, and assembly and so increasing our knowledge of these interactions may lead to increased insight into the pathology, clinical manifestations, and pathogenesis of SARS. Li *et al*. reported in 2003 that angiotensin-converting enzyme 2 acted as a functional receptor for the SARS-CoV by binding to SARS-CoV S protein [20]. To further examine the role of such interactions in the pathogenesis of SARS-CoV infection, we investigated the ability of SARS-CoV nucleocapsid protein to bind to host cell proteins. The prokaryotic expression vector pET22b was used for soluble expression of SARS-CoV NP. This vector contains the PeIB signal peptide to assure correct conformation of NP in *E*. *coli*. Because the SARS-CoV NP, unlike other SARS-CoV proteins, contains no glycosylation site, the recombinant form shows identical immune reactivity to that of the native protein [21].

Data suggested that the proteasome subunit p42 of 2BS cells interacted with SARS-CoV NP. To our knowledge, this is the first report of such an interaction. Proteasome subunit p42 (also called the 26S protease regulatory subunit S10B) is a subunit of PA700 in the ubiquitin-proteasome pathway (UPP). This pathway is one of a number of intracellular proteolytic systems in eukaryotes and mediates ATP-dependent degradation of ubiquitinated proteins. The 26S ATP-dependent proteasome, formed from the 20S proteasome and 19S regulatory particle (PA700), is a multi-protein complex. The 20S component has proteolytic activity, while the PA700 component binds ubiquitinated proteins and promotes their degradation by the 20S component. Consistent with its ATPase activity, the proteasome subunit p42 contains an ATP binding region in a conserved 200 aa domain. The proteasome subunit p42, together with the five other subunits, TBP1, MSS1, S4, p45, and TBP7, assemble to form the PA700 component. 26S proteasomes are distributed in the cytoplasm and nucleolus [22,23]. The ubiquitin-proteasome pathway mediates turnover of intracellular proteins. It plays a central role in the degradation of short-lived and regulatory proteins that are themselves vital for correct functioning of a variety of basic cellular processes including regulation of the cell cycle, modulation of cell surface receptor and ion channel expression, and antigen processing and presentation [24].

While proteasomes do hydrolyze endogenous proteins, they are also capable of degrading exogenous proteins: for example, viral peptides. In this way, these complexes are involved in defenses against viral infection. Interactions between viruses and UPP have recently been reported. Hepatitis B virus X protein (HBX) has been demonstrated to target the proteasome complex after viral entry into the cell [25]. Hu revealed that HBX is both a substrate and a potential inhibitor of the proteasome complex. The authors suggested that HBX may inhibit proteasome-mediated proteolysis by binding two subunits (Sub4, Sub7) of the 26S proteasome [26]. HBX expression inhibited turnover of c-Jun and ubiquitin- arginine-β-galactosidase, both known substrates of UPP [27]. HIV1 Tat protein was shown to inhibit the degradative activity of 20S proteasomes, by interacting with the S6a subunit of the 26S component [28]. Adenovirus E1A protein enhanced degradation of topoisomerase II alpha protein, via interaction with the S8 subunit of the 26S component [29].

Evidence to date suggests that viruses employ two strategies for proteasome inhibition; first, interference with the antigen presentation activity of the proteasome, thus promoting immune evasion. Second, stimulation of the degradative activity of the 26S proteasome causes an acceleration of G1/S turnover, thus promoting viral replication by interference with the cell cycle [30]. During viral infection, UPP hydrolyzes viral proteins into short peptides, which are then presented to CTL via MHC-1. Activated CTL then attack virally infected cells. The transcription factor nuclear factor-κB (NF-κB) is involved in regulation of immunity and inflammation; the UPP plays a central role in the regulation of NF-κB activation. When a cell is infected by a virus, IκB is phosphorylated and hydrolyzed by UPP, thus releasing NF-κB, which enters the nucleus and activates transcription of genes encoding inflammatory responses [31]. Thus, the UPP may play a vital role in infection, and the host response to infection, by a variety of viral pathogens. Development of a specific inhibitor of UPP may have potential not only as an antiviral, but also as an anti-tumor and anti-inflammatory agent. Study of the interactions of components of the UPP with viral proteins may provide useful information relating to the pathogenesis of a number of diseases [32].

Determining potential mechanisms of the interaction of SARS-CoV NP with proteasome subunit p42 was outside the scope of this study. However, such information may enhance our understanding of the activities of proteasomes in eukaryotic cells. We assumed that the interaction between SARS-CoV NP and proteasome subunit p42 inhibited the proteolytic activity of proteasomes. The actions of SARS-CoV NP and some other viral proteins may act to increase the deleterious effect of SARS-CoV infection, resulting in increased permeability, inflammatory infiltration, and mononuclear cell soakage, perhaps even accelerating pulmonary fibrosis. Moreover, this interaction may impair proteolysis of viral proteins and their presentation to CTLs and thus aid SARS-CoV evasion of immune effectors. Furthermore, such activity may affect inflammatory processes, resulting in the immunopathological damage so common in SARS. SARS-CoV NP is post-translationally modified by covalent attachment to small ubiquitin-like modifiers. Sumoylation of NP aids in promoting homo-oligomerization and assists in interference with host cell division [33].

Whether SARS-CoV NP interacts with proteasome subunit p42 *in vivo *remains unknown, and so further studies are required to fully determine the nature of this interaction and its effect *in vivo *during SARS-CoV infection. Furthermore, more research on the downstream effect of this interaction may lead to the development of novel antiviral agents effective against this debilitating and often fatal infection.

## Conclusions

In this study, we demonstrated for the first time that SARS-CoV NP interacts with the proteasome subunit p42 *in vitro*. This finding may lead to a new direction in research focusing on the molecular mechanisms of SARS-CoV infection and the pathogenesis of SARS. Whether the interaction of SARS-CoV NP and p42 impacts presentation of viral antigens and assists in viral evasion of CTLs and/or promotes enhanced inflammatory responses resulting in immunopathological damage remain to be determined.

## Abbreviations

RNA: Ribonucleic Acid; PCR: Polymerase Chain Reaction; ELISA: Enzyme-Linked Immunosorbent Assay; DNA: Deoxyribonucleic Acid; mAb: Monoclonal Antibody; HRP: Horseradish Peroxidase; TMB: Tetramethyl Benzidine; HPLC: High Performance Liquid Chromatography; EDTA: Ethylenediamine Tetraacetic Acid; IFN: Interferon; IL: Interleukin; TNF: Tumor Necrosis Factor; MIP: Macrophage Inflammatory Protein; IP: Interferon-inducible Protein; ORF: Open Reading Frame; ATP: Adenosine Triphosphate; HBX: X protein of HBV; CTL: Cytotoxic T-Lymphocyte; MHC: Major Histocompatibility Complex.

## Competing interests

The authors declare that they have no competing interests.

## Authors' contributions

QW and DL participated in the design and conducted the majority of the experiments in the study and drafted the manuscript. TW and JL contributed to the interpretation of the findings and revised the manuscript. QZ and CL carried out ELISA tests. ML edited the manuscript. JY and WG performed analyses of data. All authors read and approved the final manuscript.
